# ALS-linked misfolded SOD1 species have divergent impacts on mitochondria

**DOI:** 10.1186/s40478-016-0313-8

**Published:** 2016-04-27

**Authors:** Sarah Pickles, Sabrina Semmler, Helen R. Broom, Laurie Destroismaisons, Laurine Legroux, Nathalie Arbour, Elizabeth Meiering, Neil R. Cashman, Christine Vande Velde

**Affiliations:** Centre de recherche du Centre Hospitalier de l’Université de Montréal (CRCHUM) Université de Montréal, 900 rue Saint-Denis, Local R09.442, Montréal, QC H2X 0A9 Canada; Department of Biochemistry, Université de Montréal, Montréal, QC H2X 0A9 Canada; Department of Neurosciences, Université de Montréal, Montréal, QC H2X 0A9 Canada; Integrated Program in Neuroscience, McGill University, Montréal, QC H3A 2B4 Canada; Department of Biochemistry, University of Waterloo, Waterloo, ON N2L 3G1 Canada; Department of Medicine (Neurology), University of British Columbia and Vancouver Coastal Health Research Institute, Brain Research Centre, Vancouver, BC V6T 2B5 Canada

**Keywords:** Amyotrophic Lateral Sclerosis, Mitochondria, Superoxide dismutase, Flow cytometry

## Abstract

Approximately 20 % of familial Amyotrophic Lateral Sclerosis (ALS) is caused by mutations in superoxide dismutase (*SOD1)*, which leads to misfolding of the SOD1 protein, resulting in a toxic gain of function. Several conformation-restricted antibodies have been generated that specifically recognize misfolded SOD1 protein, and have been used as therapeutics in pre-clinical models. Misfolded SOD1 selectively associates with spinal cord mitochondria in SOD1 rodent models. Using the SOD1^G93A^ rat model, we find that SOD1 conformational specific antibodies AMF7-63 and DSE2-3H1 labeled a fibrillar network concentrated in the anterior horn; while A5C3, B8H10, C4F6 and D3H5 labeled motor neurons as well as puncta in the neuropil. There is a time-dependent accumulation of misfolded SOD1 at the surface of spinal cord mitochondria with AMF7-63-labeled mitochondria having increased volume in contrast to a mitochondrial subset labeled with B8H10. In spinal cord homogenates and isolated mitochondria, AMF7-63, DSE2-3H1 and B8H10 detect misfolded SOD1 aggregates. SOD1 that lacks its metal cofactors has an increased affinity for naïve mitochondria and misfolded SOD1 antibodies B8H10 and DSE2-3H1 readily detect demetalated mutant and wild-type SOD1. Together, these data suggest that multiple non-native species of misfolded SOD1 may exist, some of which are associated with mitochondrial damage. Conformational antibodies are invaluable tools to identify and characterize the variation in misfolded SOD1 species with regards to biochemical characteristics and toxicity. This information is highly relevant to the further development of these reagents as therapeutics.

**Electronic supplementary material:**

The online version of this article (doi:10.1186/s40478-016-0313-8) contains supplementary material, which is available to authorized users.

## Introduction

The defining feature of the neurodegenerative disease Amyotrophic Lateral Sclerosis (ALS) is the loss of motor neurons in the cortex, brain stem and spinal cord [1]. Loss of motor neurons leads to denervation resulting in muscle weakness, atrophy and eventual paralysis. Despite identification of the first gene linked to familial ALS (FALS), Superoxide Dismutase 1 (SOD1) [2] over twenty years ago, and the discovery of many more ALS genes since, the causes of motor neuron degeneration remain unknown.

Mutations in SOD1 account for 15 to 20 % of all FALS cases, and approximately 3 % of sporadic ALS (SALS) cases [3]. SOD1 mutations universally lead to conformation changes within the native protein structure, resulting in the acquisition of an elusive toxic function [4]. Several antibodies have been developed to specifically target these altered conformations, which are collectively referred to as misfolded SOD1 (reviewed in [5, 6]). Recombinant SOD1^G93A^ protein lacking its metals (apo), including the structure stabilizing zinc cofactor, was used for immunization which led to the generation of a heterogeneous pool of antibodies with different affinities and reactivity to distinct epitopes located on one or more of the SOD1^G93A^ protein conformers. These antibodies were subsequently clonally expanded to monoclonal antibodies named as A5C3, B8H10, C4F6, and D3H5 [7, 8]. Other antibodies, such as DSE2-3H1, SEDI, USOD, and a series of polyclonal antibodies produced by Forsberg and colleagues, were produced via immunization with peptides comprised of amino acids that are normally inaccessible in the well folded protein [9–11]. All of these antibodies recognize epitopes that are exposed only when SOD1 adopts a non-native conformation induced either by mutation, loss of its zinc cofactor, and/or oxidation. While many of these were developed with the intent to be potential therapeutics, these reagents have also become valuable tools with which to track the toxic forms of SOD1. Misfolded SOD1 is detected predominantly within the motor neurons of ALS animal models [8, 11–14]. In humans, various antibodies report on misfolded SOD1 in neurons of FALS patients as well as SALS patients, although this latter finding remains controversial [7, 9, 15, 16]. In pre-clinical research using mutant SOD1 animals, it is now appreciated that reducing misfolded SOD1 levels via immunization significantly increases survival [8, 17]. This provides additional support that misfolded SOD1 lies at the root of SOD1-mediated ALS [8, 17].

Despite consensus in the field that misfolded SOD1 is central to disease pathogenesis, it remains unknown how misfolded SOD1 causes motor neuron death. Misfolded SOD1 has been implicated in the induction of endoplasmic reticulum (ER) stress [12, 18], defective axonal transport [7], alteration of motor neuron excitability [19], and mitochondrial dysfunction [11, 13, 14, 20] in SOD1-mediated ALS disease models. Multiple aspects of mitochondrial physiology are disrupted in mutant SOD1 cell culture and animal models including morphology [21–23], adenosine triphosphate (ATP) generation [24], calcium handling [25], axonal transport [26] and protein import [27]. Interestingly, misfolded SOD1 directly associates with mitochondria derived from affected, but not unaffected tissues [11]. The selective association of misfolded SOD1 to spinal cord mitochondria has just recently been attributed to a lack of the putative chaperone macrophage migration inhibitory factor (MIF) in this tissue, and more specifically motor neurons [28].

Recent evidence suggests that multiple non-native/misfolded SOD1 species may exist [29–31]. Consistent with this concept, we have previously reported that the B8H10 antibody detects misfolded SOD1 in both cytosolic and mitochondrial fractions prepared from SOD1^G93A^ spinal cords while the C4F6 antibody exclusively detects cytosolic misfolded SOD1 [13]. Other work in cultured cells made to overexpress mutant SOD1 indicates that the C4F6 antibody recognizes soluble mutant protein, whereas SEDI preferentially detects mutant SOD1 within inclusions [31]. Additionally, a series of polyclonal SOD1 peptide-specific antibodies identify two different forms of SOD1 aggregates (or “strains”) in mutant SOD1 mice based on epitope accessibility, with one such aggregate-type/strain correlating with an earlier age of onset [30]. Together these data suggest that multiple forms of misfolded SOD1 are possible.

We hypothesized that if more than one form of misfolded SOD1 exists, there may be conformer-specific differences in localization, potency and/or pathomechanistic consequences. To this end, we have employed a panel of misfolded SOD1-specific antibodies, to evaluate misfolded SOD1 localization, ability to induce mitochondrial toxicity and incorporation into aggregates. Herein, we report that the misfolded SOD1-specific antibody DSE2-3H1 labels motor neurons and robustly detects fibrils in the anterior horn of SOD1^G93A^ spinal cords, a finding that is confirmed by a second independent antibody raised against the same peptide immunogen (AMF7-63). Other misfolded SOD1-specific antibodies, A5C3, B8H10, C4F6 and D3H5 antibodies label predominantly to motor neurons and numerous neuropil puncta. Despite their different labeling patterns within the spinal cord, both B8H10 and AMF7-63 antibodies immunolabel spinal cord mitochondria in a time-dependent manner. However, the presence of AMF7-63-reactive misfolded SOD1 at mitochondria correlates with a more severe dysregulation of mitochondrial volume compared to mitochondria without associated misfolded SOD1.

## Materials and methods

### Animals

SOD1^G93A^ and SOD1^WT^ transgenic rats have been previously described [32, 33]. Non-transgenic littermates were used in some experiments. Early symptomatic is defined as animals that have a noticeable gait defect, hopping or limping, typically involving only one limb. Both male and female rats were used. Animals were treated in strict adherence to approved protocols from the CRCHUM Institutional Committee for the Protection of Animals and the Canadian Council on Animal Care (CCAC).

### Antibodies

Rabbit anti-Cu/Zn SOD (Enzo Life Sciences), rabbit anti-SOD1 (Cell Signaling), mouse anti-VDAC1 (Calbiochem), mouse anti-Actin (MP Biomedicals), were used for immunoblots. Anti-misfolded SOD1 mouse monoclonal antibodies D3H5 (1:250, generously provided by Dr. J-P Julien), A5C3 (1:50), B8H10 (1:250) and C4F6 (1:250) (Medimabs), DSE2-3H1 (1:1000), rabbit monoclonal antibody AMF7-63 (1:1500) and rabbit polyclonal antibody SEDI (1:100, generously provided by Dr. J. Robertson) were used for immunoblotting, immunofluorescence and flow cytometry. Mouse and rabbit IgG (Jackson ImmunoResearch Labs) and mouse anti-IgG1 (BD Biosciences) were used as controls. Goat anti-mouse allophycocyanin-conjugated (BD Pharmingen), goat anti-rabbit PE (eBioscience) and goat anti-rabbit PE-Cy7-conjugated (Santa Cruz) secondary antibodies were used for flow cytometry studies. For immunofluorescence, goat anti-ChAT (1:100; Millipore), mouse anti-SMI32 (1:2000; Covance), mouse anti-SMI31 (1:2000; Covance) and mouse anti-MAP2 (1:500; Sigma) were used.

### Flow cytometry of isolated mitochondria

Spinal cord and liver mitochondria were isolated from mice and rats [11], and prepared for analysis by flow cytometry, as previously described [13, 34].

### Immunoprecipitation and immunoblotting

Isolated mitochondria were solubilized and immunoprecipitated as previously described [11]. Briefly, 50 μg of mitochondria were incubated with 15 μL Protein G magnetic beads (Invitrogen), overnight at 4 °C with rotation. Protein G beads were previously incubated with misfolded SOD1-specific antibody. Immunoprecipitated proteins were eluted from the beads in 2.5× Laemmli buffer and electrophoresed on 15 % Tris-Glycine gels, and subsequently transferred to nitrocellulose.

### Immunofluorescence

Sections were labeled with anti-misfolded SOD1 antibodies, as previously described [13]. Briefly, sections were washed 10 min at room temperature in PBS, then permeabilized for 10 min at room temperature in PBS with 0.4 % TX-100. Sections were blocked with 2 % normal donkey serum (Sigma), 2 % bovine serum albumin (Sigma), in 0.4 % TX-100/PBS for 1 h at room temperature. Primary antibodies were incubated overnight at 4 °C in blocking solution. Appropriate secondary antibodies were added in blocking solution for 1 h at room temperature. Sections were mounted using ProLong antifade reagent (Invitrogen). Immunofluorescent images were captured by confocal microscopy (Leica SP5; 20× and 40× objective, 1.7 NA) and processed with Leica LAS AF software and/or PhotoshopCS4 (Adobe).

### Filter-trap assay

20 μg of spinal cord homogenates or isolated spinal cord mitochondria in PBS were filtered through a 0.22 μm cellulose acetate membrane (GE Healthcare) using the Bio-Dot Microfiltration Apparatus (Bio-Rad). Wells were washed twice with PBS, the membrane was removed from the apparatus and then blocked 1 h at room temperature and immunoblotted with misfolded SOD1-specific antibodies. Mitochondria for these experiments were prepared by floating upwards to their buoyant density so as to avoid possible co-sedimentation of aggregates, as previously described [11].

### Dot blot of recombinant SOD1 protein

1 μg of recombinant SOD1 protein, produced as previously described [35–37], in TBS (20 mM Tris, 500 mM NaCl, 1 mM EDTA pH 7.5) was spotted onto nitrocellulose membrane (BioRad) using the Bio-Dot Microfiltration Apparatus (Bio-Rad). Wells were washed twice with TBS, and the membrane was removed from the apparatus and blocked in TBS-T (as above plus 0.05 % Tween-20) with 1 % bovine serum albumin (BSA) for 30 min at room temperature, and immunoblotted with misfolded SOD1 antibodies. Primary and secondary antibodies were incubated in blocking buffer. For non-native samples, 5 % v/v BME, and 0.5 % v/v SDS was added, and samples were heat denatured by incubation for 5 min at 95 °C.

### In vitro mitochondrial binding assay

50 μg of isolated spinal cord mitochondria (2 μg/μL) from non-transgenic rats were incubated with 3 μM baculovirus-produced SOD1^WT^ and SOD1^G93A^ recombinant protein, purified as previously described [38], for 20 min at 37 °C in HB Buffer (210 mM Mannitol, 70 mM Sucrose, 10 mM Tris pH 7.5, 1 mM EDTA) [38]. Mitochondria were washed once with HB buffer and then re-suspended in HB and 4× Laemmli sample buffer and subjected to SDS-PAGE and immunoblotted with an antibody to human SOD1 (Cell Signaling). To determine if modification of SOD1 structure would alter its binding to the mitochondrial surface, the protein was incubated with 5.5 mM EDTA or 10 mM hydrogen peroxide in PBS overnight at 4 °C or room temperature, respectively, with protease inhibitors (Roche). EDTA and hydrogen peroxide were removed and replaced by PBS by dialysis with Slide-A-Lyzer Mini dialysis devices (Pierce). Untreated samples were treated equivalently.

### Statistics

Two-way ANOVA was used to determine the interaction between groups and time for percentage of misfolded SOD1^+^ mitochondrial subpopulations over time and differences in AMF7-63^+^, B8H10^+^, and negative mitochondrial subpopulations over time. Sidak’s multiple comparison test was used to determine differences between misfolded SOD1^+^ groups. One-way ANOVA was used to determine differences in AMF7-63^+^, AMF7-63^+^B8H10^+^ and B8H10^+^ subpopulations. * *P* < 0.05, ** *P* < 0.01 *** *P* < 0.001, **** *P* < 0.0001. All analyses was done with GraphPad Prism software.

## Results

### Misfolded SOD1 specific antibodies DSE2-3H1 and AMF7-63 detect fibrils in the spinal cord of SOD1^G93A^ rats

**Fig. 1:**
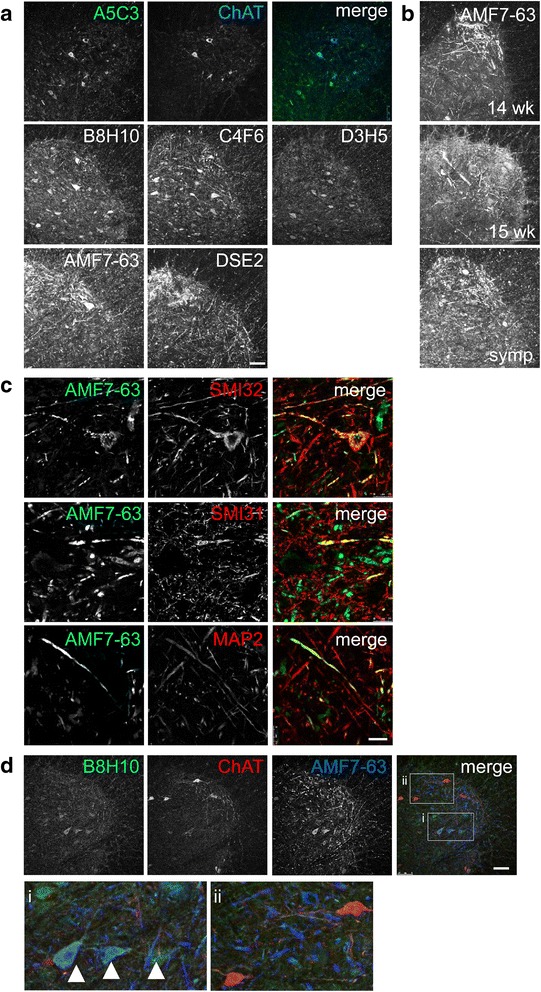
Misfolded SOD1-specific antibodies have distinct labeling patterns in SOD1^G93A^ rat spinal cords. Immunohistochemistry for misfolded SOD1 in SOD1^G93A^ lumbar spinal cords. **a** Lumbar sections of a symptomatic SOD1^G93A^ rat were stained with misfolded SOD1 specific antibody A5C3 (*green*) and co-labeled with ChAT (*blue*). Additional representative images of B8H10, C4F6, D3H5, DSE2-3H1 and AMF7-63 are also shown. **b** The AMF7-63 antibody detects fibrils in pre-symptomatic, 14 and 15 week SOD1^G93A^ rat spinal cords. **c** Symptomatic SOD1^G93A^ rat spinal cord was labeled with AMF7-63 (*green*) and SMI32 (red), SMI31 (*red*), or MAP2 (*red*). **d** Lumbar sections labeled with misfolded SOD1 antibodies AMF7-63 (*blue*), B8H10 (*green*), and co-labeled with ChAT (*red*). Two to three animals of each genotype were analyzed. Scale bar = 100 μm for (**a**, **b** and **d**) and 25 μm for (**c**)

To evaluate whether multiple antibodies targeted to non-native/misfolded conformations of SOD1 yielded a universal localization under similar conditions, lumbar sections of symptomatic SOD1^G93A^ rat spinal cords were labeled with a panel of misfolded SOD1 specific antibodies. In symptomatic SOD1^G93A^ rats, all of the misfolded SOD1-specific antibodies tested (A5C3, B8H10, C4F6, D3H5, DSE2-3H1, and AMF7-63) labeled motor neurons as marked by choline acetyltransferase (ChAT) (Fig. 1a). Antibodies DSE2-3H1 (mouse monoclonal) and AMF7-63 (rabbit monoclonal) were raised against the same epitope located in the electrostatic loop (amino acids ^125^DDLGKGGNEESTKTGNAG^142^) of SOD1 which is normally inaccessible in the well-folded SOD1 structure [11]. In symptomatic animals, both antibodies intensely labeled the anterior horn albeit with varying affinities, with AMF7-63 demonstrating a more intense labeling (Fig. 1a). This is consistent with a 10^3^-fold enhanced affinity for the immunogenic peptide (N. Cashman, unpublished data). The labeling resembled a network of fibril-like structures, with some motor neuron soma being obviously labeled. In general, the fibrillar pattern detected by DSE2-3H1 and AMF7-63 was so robust, it was often difficult to discern individual motor neurons. These two antibodies also revealed a fibrillar network in pre-symptomatic animals (14 and 15 weeks old, Fig. 1b). In contrast, fibrils were only occasionally detected in sections labeled with misfolded SOD1 antibodies A5C3, B8H10 and C4F6, but not D3H5, in pre-symptomatic as well as symptomatic animals (data not shown). Instead, we noted that A5C3, B8H10, C4F6 and D3H5 antibodies homogenously labeled motor neuron somata, and this was accompanied by numerous small puncta observed throughout the neuropil (Fig. 1a). As expected, none of the antibodies yielded a signal in lumbar spinal cords of age-matched rats expressing comparable levels of human wild type SOD1 (SOD1^WT^), thereby confirming antibody specificity, nor was there non-specific labeling in IgG controls or sections stained with secondary antibody alone (Additional file 1: Figure S1A, B). AMF7-63-reactive misfolded SOD1 fibrils could be localized to different motor neuron subcompartments including the soma, dendrites (SMI32, MAP2) and axons (SMI31) (Fig. 1c). These initial studies indicate that misfolded SOD1-specific antibodies can be broadly considered as two distinct groups: A5C3, B8H10, C4F6, and D3H5 which label motor neurons and numerous puncta throughout the neuropil; and AMF7-63 and DSE2-3H1 which also label motor neurons but also intensely reveal fibril-like structures. Based on these spatial considerations, these data suggest that more than one type of misfolded SOD1 species exists in vivo.

To determine if these seemingly different misfolded SOD1 conformations could co-exist within the same motor neuron, spinal cord sections were co-labeled with AMF7-63 and B8H10. A partial co-localization of these two antibodies within ChAT-positive motor neurons was frequently observed (Fig. 1d), suggesting that these antibodies recognize apparently distinct non-native SOD1 species within the same neurons. In addition, we observed neurons that labeled with AMF7-63 uniquely (ie. void of B8H10), and *vice versa*.

### AMF7-63 antibody detects misfolded SOD1^G93A^ at the mitochondrial surface

**Fig. 2:**
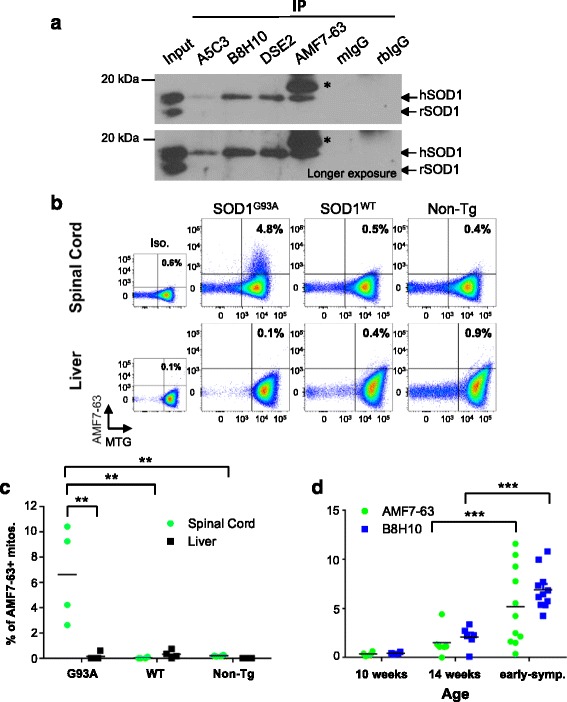
B8H10 and AMF7-63 reactive misfolded SOD1 is present in SOD1^G93A^ spinal mitochondrial fractions. **a** Immunoprecipitation for misfolded SOD1 in isolated SOD1^G93A^ mitochondria with A5C3, B8H10, DSE2 3H1 and AMF7-63. Mouse (mIgG) and rabbit IgG (rbIgG) serve as controls. Input is 10 μg isolated mitochondria. Upper bands and lower bands correspond to human (hSOD1) and rat (rSOD1) SOD1, respectively. Immunoprecipitation with AMF7-63 resulted in a non-specific band (*) just above human SOD1, regardless of misfolded SOD1 status. Experiment shown is representative of three independent trials. **b** Immunolabeling of isolated spinal cord and liver mitochondria with misfolded SOD1-specific antibody AMF7-63 from symptomatic SOD1^G93A^ rats and controls (aged matched SOD1^WT^ and non-transgenic rats) by flow cytometry. Misfolded SOD1 positive labeling is determined by comparing to isotype control (rabbit IgG, dotplots in first column) of SOD1^G93A^ sample. Percentage of misfolded SOD1^+^ events is shown for each tissue and genotype in a representative sample. **c** Quantification of AMF7-63^+^ events in spinal cord (*green circle*) or liver (*black square*) of symptomatic SOD1^G93A^ rats, and age- matched SOD1^WT^ as well as non-transgenic rats. Data are represented as percent of misfolded AMF7-63^+^ mitochondria, each dot represents one animal, *n* = 4 animals per genotype per tissue. **d** Comparison of spinal cord mitochondrial labeling positive for AMF7-63 (*green, circle*) or B8H10 (*blue, square*) in pre-symptomatic (10 and 14 weeks) and symptomatic SOD1^G93A^ rats by flow cytometry. Data are represented as percentage of misfolded SOD1^+^ mitochondria, each dot represents one animal, (mean), *n* = 4–11 animals. ** *P* < 0.01. *** *P* < 0.001

Several misfolded SOD1-specific antibodies (DSE2-3H1, A5C3, B8H10, SEDI) recognize misfolded SOD1 protein deposited on the cytoplasmic face of the mitochondrial outer membrane [10, 11, 13, 14, 20]. However, this is not a universally shared feature of misfolded SOD1 as conformers recognized by C4F6 are primarily cytosolic and exhibit little to no mitochondrial association [13]. We sought to determine if AMF7-63-reactive misfolded SOD1 also associates with mitochondria. Misfolded SOD1-specific antibodies A5C3, B8H10, DSE2-3H1 and AMF7-63 were used to immunoprecipitate misfolded SOD1 from spinal cord mitochondria isolated from symptomatic SOD1^G93A^ animals (Fig. 2a). As previously published, B8H10 and DSE2-3H1-reactive SOD1 were robustly detected in mitochondrial fractions [11, 13, 28] (Fig. 2a). AMF7-63 detected similar amounts of misfolded SOD1 (Fig. 2a). A5C3, which we have previously demonstrated to label misfolded SOD1 on distal axonal mitochondria [11] also detected misfolded SOD1 within mitochondrial fractions, but to a lesser extent than the other antibodies (Fig. 2a). While a non-specific band migrating just above SOD1 was detected when the AMF7-63 antibody was used for immunoprecipitation, the specificity of the AMF7-63 antibody for misfolded SOD1 was confirmed by immunoprecipitation of spinal cord homogenates and isolated mitochondria from symptomatic SOD1^G93A^ rats as well as age-matched SOD1^WT^ rats. As expected, AMF7-63 immunoprecipitated misfolded SOD1 exclusively from SOD1^G93A^ rat spinal cord homogenates and mitochondria, but not similar preparations from livers or SOD1^WT^ tissue (Additional file 2: Figure S2).

We wondered if the presence of AMF7-63-reactive misfolded SOD1 conformer negatively impacted mitochondrial function. To this end, we employed surface-labeling with the misfolded SOD1-specific antibodies AMF7-63 and B8H10 and subsequent flow cytometric detection of isolated spinal cord mitochondria [34]. Using early symptomatic SOD1^G93A^ rats, we established that AMF7-63 preferentially detected misfolded SOD1 on isolated spinal cord mitochondria compared to liver, a tissue that is unaffected in ALS (Fig. 2b). Significantly more individual spinal cord mitochondria (as marked by the indicator dye MitoTracker Green, MTG) from SOD1^G93A^ rats labeled for AMF7-63 (6.6 ± 1.9 %) compared to SOD1^WT^ (0.1 ± 0.03 %) and non-transgenic (0.2 ± 0.03 %) animals (SOD1^G93A^ vs, SOD1^WT^, non-transgenic: *P* < 0.001) (Fig. 2b, c). As expected, AMF7-63-reactive misfolded SOD1 was not detected (ie. below 1 %) on liver mitochondria from any group, confirming specificity of misfolded SOD1 for affected tissues (SOD1^G93A^: 0.1 ± 0.1 %; SOD1^WT^: 0.3 ± 0.02 %; non-transgenic: 0.1 ± 0.2 %, *n* = 4 animals per genotype) (Fig. 2c). Similarly, and consistent with our previous work [13], B8H10^+^ mitochondria were robustly detected in mitochondrial preparations from symptomatic SOD1^G93A^ spinal cords (6.1 ± 0.4 %) but not SOD1^WT^ (0.4 ± 0.2 %) or non-transgenic cords (0.4 ± 0.1 %) (SOD1^G93A^ vs, SOD1^WT^, non-transgenic: *P* < 0.0001, *n* = 3 animals per genotype) (Additional file 3: Figure S3A, B). There was no substantial B8H10 labeling of liver mitochondria in any animal model tested (Additional file 3: Figure S3A, B).

At an early symptomatic stage, surface labeling of isolated spinal cord mitochondria demonstrated that both AMF7-63 and B8H10 antibodies detected misfolded SOD1 at the cytoplasmic face of the mitochondrial outer membrane (Fig. 2d). Thus, we asked whether there was a temporal difference in the accumulation of these two conformers. Spinal cord mitochondria from 10 week old, 14 week old and early symptomatic SOD1^G93A^ rats were processed for labeling with B8H10 and AMF7-63. While no mitochondrial signal for either antibody was detected at 10 weeks, comparable labeling was detected in mitochondria from 14 week animals (AMF7-63^+^: 1.5 ± 0.6 %; B8H10^+^: 2.1 ± 0.5 %). Higher proportions of mitochondria labeled for misfolded SOD1 at the early symptomatic stage compared to the 10 and 14 week groups, demonstrating a significant age-dependent accumulation of each misfolded SOD1 conformer at the mitochondrial surface (*P* < 0.0001). Comparison of the relative amounts of AMF7-63^+^ (5.2 ± 1.1 %) and B8H10^+^ (6.9 ± 0.6 %) subpopulations yielded no significant differences (Fig. 2d). Collectively, these data would suggest that there is no preferential temporal accumulation of these two forms of non-native SOD1 conformers at the mitochondrial surface.

**Fig. 3:**
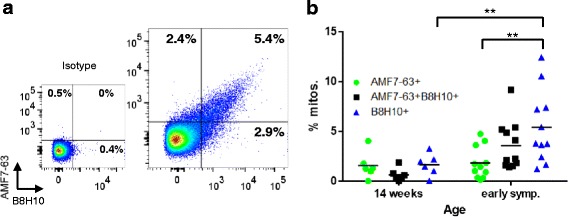
Four distinct mitochondrial subpopulations revealed by simultaneously immunolabeling with AMF7-63 and B8H10 misfolded SOD1-specific antibodies. **a** Flow cytometric analysis of isolated spinal cord mitochondria from symptomatic SOD1^G93A^ animals labeled with AMF7-63 and B8H10. Analysis only includes events previously gated for MTG staining (MTG^+^). Percentage of misfolded SOD1^+^ events is shown for each subpopulation in a representative experiment. **b** Quantification of AMF7-63^+^B8H10^−^ (*green, circle*), AMF7-63^+^B8H10^+^ (*black, square*) and B8H10^+^ AMF7-63^−^ (*blue, triangle*) mitochondrial subpopulations from spinal cords of 14 week and early symptomatic SOD1^G93A^ rats. Data are represented as percent of misfolded SOD1^+^ mitochondria (mean), *n* = 4–11 animals per subpopulation. ** *P* < 0.01

Given these latter data, one could argue that the antibodies are detecting the same conformer in vivo. Thus, to address this, we analyzed our data to determine if it was possible to detect B8H10-labeled mitochondria void of AMF7-63 labeling, and *vice versa*. By simultaneously immunolabeling isolated mitochondria with both AMF7-63 and B8H10, we were able to discern four distinct mitochondrial subpopulations: *i)* double negative (AMF7-63^−^B8H10^−^); *ii)* AMF7-63 only (AMF7-63^+^B8H10^−^) 2.4 % in the illustrated example; *iii)* B8H10 only (AMF7-63^−^B8H10^+^), 2.9 % in the illustrated example; and *iv)* double positive (AMF7-63^+^B8H10^+^), 5.4 % in the illustrated example (Fig. 3a). Therefore, the total AMF7-63^+^ subpopulation (7.8 %) consists of both B8H10^+^ (5.4 %) and B8H10^−^ mitochondria (2.4 %). That a proportion of mitochondria label positive with both misfolded SOD1 antibodies, while others label for only one conformer, suggests that AMF7-63 and B8H10 recognize distinct misfolded SOD1 species.

From 14 weeks to the early symptomatic stage, there was a significant time dependent increase in the percentage of misfolded SOD1 B8H10-labeled mitochondria (*P* < 0.01) (Fig. 3b). The relative amounts of the three subpopulations with surface-bound misfolded SOD1 revealed no significant differences between them at 14 weeks. Interestingly, between 14 weeks and the early symptomatic stage, the proportion of AMF7-63^+^B8H10^+^ and AMF7-63^−^B8H10^+^ mitochondrial subpopulations nearly doubled, whereas the AMF7-63^+^ B8H10^−^ subpopulation remained roughly constant (Fig. 3b). At the latter time point, there is a significantly higher percentage of AMF7-63^−^B8H10^+^ than AMF7-63^+^B8H10^−^ mitochondria (*P* < 0.01) (Fig. 3b). These data suggest there is either preferential removal of the AMF7-63 only subpopulation or disturbed removal/enhanced accumulation of B8H10 only mitochondria.

### Volume dyshomeostasis and superoxide production is enhanced in mitochondria with surface-bound AMF7-63-reactive SOD1

**Fig. 4:**
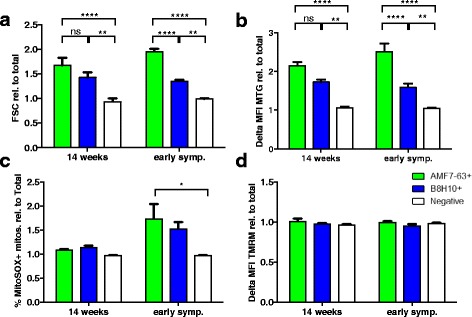
Presence of AMF7-63 reactive misfolded SOD1 at the mitochondrial surface coincides with increased mitochondrial volume. Flow cytometric analysis of isolated spinal cord mitochondria from 14 week and early symptomatic SOD1^G93A^ rats labeled with misfolded SOD1 specific antibodies AMF7-63 and B8H10. Analysis includes only events previously gated for MTG staining (MTG^+^). **a** Quantification of the geometric mean of FSC of AMF7-63^+^ (*green*) B8H10^+^ (*blue*) and negative (*white*) mitochondrial subpopulations relative to total population in 14 week and symptomatic SOD1^G93A^ rats. **b** Quantification of delta mean fluorescence intensity (ΔMFI) of MTG staining of mitochondrial sub-populations relative to total population. **c** Quantification of percentage of MitoSOX^+^ mitochondria from mitochondrial subpopulations relative to total population. Each subpopulation was normalized for MTG staining. **d** Quantification of ΔMFI of TMRM staining of mitochondrial subpopulations relative to total population and normalized to size/MTG. *n* = 4–5 animals per time point. Not significant (ns), * *P* < 0.05, ** *P* < 0.01, **** *P* < 0.0001

A distinct advantage of the flow cytometry-based method to detect mitochondrially-associated misfolded SOD1 is that it permits the simultaneous use of fluorescent indicator dyes to report on mitochondrial functions. Since a difference in the timing of mitochondrial association between AMF7-63^+^ and B8H10^+^ mitochondria was not detected (Fig. 2d), we hypothesized that perhaps they may exhibit variable toxicity towards mitochondria. Thus, we evaluated select aspects of mitochondrial function in the AMF7-63^+^ subpopulation. Mitochondrial size/volume was assessed by flow cytometry on the basis of the intensity of forward light scatter (FSC) of individual mitochondria [39, 40]. Quantification of the FSC normalized to the total population indicate that the total mitochondrial subset bearing AMF7-63 reactive misfolded SOD1 from 14 week and early symptomatic SOD1^G93A^ animals were significantly larger than non-coated mitochondria (*P* < 0.001) (Fig. 4a). Intriguingly, the AMF7-63^+^ subpopulation was also significantly larger than the B8H10^+^ subpopulation (regardless of AMF7-63 status) when animals began exhibiting early symptoms (*P* < 0.0001) (Fig. 4a).

Mitotracker Green (MTG) can be used not only to identify mitochondria, but also to report on mitochondrial volume. Specifically, dye uptake measured by the delta mean fluorescence intensity (ΔMFI) correlates with mitochondrial volume as the dye accumulates within mitochondria independent of the mitochondrial transmembrane potential [41]. In agreement with our FSC data, AMF7-63^+^ and B8H10^+^ subpopulations have a significantly higher ΔMFI compared to uncoated mitochondria at both time points (AMF7-63^+^, *P* < 0.0001; B8H10^+^, *P* < 0.01), with AMF7-63^+^ mitochondria taking up more dye compared to B8H10^+^ mitochondria when animals are in the early symptomatic stage (*P* < 0.0001) (Fig. 4b). Together, these data indicate that the association of AMF7-63-reactive misfolded SOD1 conformers with the mitochondrial surface correlates with enlarged mitochondria.

Superoxide is produced as a natural by-product of oxidative phosphorylation [42]. We evaluated the levels of mitochondrial superoxide produced by the AMF7-63^+^ and B8H10^+^ subpopulations using MitoSOX Red, a mitochondria-specific superoxide indicator [43, 44]. Following normalization for size differences, misfolded SOD1^+^ mitochondrial subpopulations produced significantly higher levels of superoxide compared to the misfolded SOD1^−^ subpopulation at both 14 weeks and when animals began to exhibit symptoms (*P* <0.05). Further comparison revealed that while the AMF7-63^+^ and B8H10^+^ subpopulations were not significantly different from each other, the AMF7-63^+^ subpopulation was significantly increased compared to the unlabeled subpopulations at the early symptomatic stage (*P* < 0.05, Fig. 4c). These changes were independent of mitochondrial transmembrane potential (ΔΨ_m_) which was unchanged between subpopulations (Fig. 4d). Taken together, these data suggest that there could be variable mitochondrial damage associated with different conformers of misfolded SOD1, given that AMF7-63-reactive misfolded SOD1 is associated with more severe deregulation of mitochondrial volume homeostasis, while superoxide production is equivalent to B8H10-coated mitochondria.

### Misfolded SOD1 conformers are aggregated on mitochondria

**Fig. 5:**
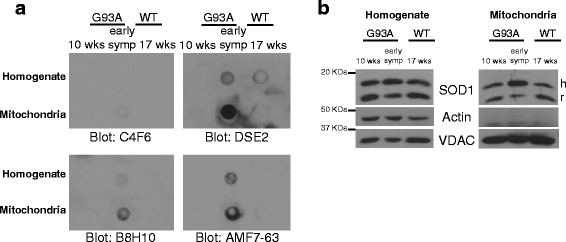
AMF7-63 and B8H10 antibodies detect aggregated misfolded SOD1 in spinal cords. **a** Size exclusion filter-trap assay of homogenates or isolated mitochondria from spinal cords of pre-symptomatic (10 week) and early symptomatic SOD1^G93A^ rats or age-matched SOD1^WT^ controls, blotted for misfolded SOD1-specific antibodies B8H10, DSE2-3H1, AMF7-63 and C4F6. Data is representative of three independent trials. **b** Homogenates and isolated mitochondria immunoblotted for SOD1 to verify expression levels. Actin and VDAC serve as loading controls

Given that B8H10 and AMF7-63-reactive misfolded SOD1 disturbed mitochondrial volume to varying degrees, we speculated this might be attributed to differences in biochemical properties. Certain misfolded SOD1-specific antibodies are reported to detect cytoplasmic aggregates/inclusions [30, 31], but whether misfolded SOD1 aggregates at/on mitochondria remains unknown. Therefore, we examined the accumulation of misfolded SOD1 into aggregates in isolated mitochondrial fractions. A filter trap assay in which proteinaceous aggregates larger than 220 nm are retained on a cellulose acetate membrane [45], was performed on homogenates and isolated mitochondria from spinal cords of pre-symptomatic (10 week) and early symptomatic SOD1^G93A^ rats as well as age-matched SOD1^WT^ animals. Given that this assay is performed in non-denaturing conditions, we reasoned that the misfolded SOD1 conformational antibodies should retain their specificity. In agreement with this, misfolded SOD1 antibodies B8H10, DSE2-3H1 and AMF7-63 preferentially labeled homogenates of SOD1^G93A^ spinal cords but not controls (Fig. 5a). Moreover, these three antibodies demonstrated more intense immunoreactivity for mitochondrial samples (which were isolated via buoyant density centrifugation so as to avoid potential co-pelleting of cytoplasmic aggregates) (Fig. 5a). Furthermore, the formation of aggregates was disease/age-dependent, with robust labeling of homogenates and isolated mitochondria from early symptomatic animals, but little to no labeling at 10 weeks. Note, the C4F6 antibody detected little to no aggregates in homogenates or isolated mitochondria at any age (Fig. 5a), consistent with reports by others that C4F6 recognizes soluble misfolded SOD1 [31, 46]. Western blots depict SOD1 expression between samples (Fig. 5b). These results suggest that misfolded SOD1 conformers recognized by B8H10, DSE2-3H1, and AMF7-63, are components of protein aggregates in both spinal cord homogenates and are enriched on mitochondria.

### Preferential recognition of demetallated and reduced recombinant SOD1

**Fig. 6:**
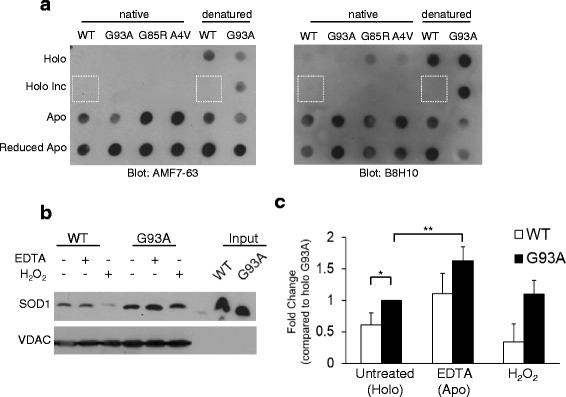
Misfolded SOD1 specific antibodies show preferential reactivity for demetallated (apo) SOD1. **a** Recombinant SOD1 proteins (WT, G93A, G85R and A4V) were produced (i) with its full complement of metals (holo); (ii) with its full complement of metals and incubated so as to produce low levels of aggregated protein (holo inc); (iii) lacking metals (apo); and (iv) lacking metals and a reduced Cys57-Cys146 disulfide bond (apo reduced) were spotted onto nitrocellulose and probed for misfolded SOD1 with AMF7-63 (*left*) and B8H10 (*right*). **b** In vitro mitochondrial binding assay. Recombinant SOD1^WT^ and SOD1^G93A^ were incubated with spinal cord mitochondria from a non-transgenic rat, washed and subjected to analysis by western blot. Recombinant SOD1 was either left untreated or incubated with EDTA or H_2_O_2_ before addition to mitochondria. **c** Quantification of **b** normalized to SOD1^G93A^ binding SOD1^WT^ (*white*) and SOD1^G93A^ (*black*). * *P* < 0.05, ** *P* < 0.01, *n* = 5

To determine if the misfolded SOD1-specific antibodies have a particular affinity to certain gross perturbations in SOD1 structure, demetallation, aggregation or reduction of the intra-molecular disulfide bond, recombinant wild-type and various SOD1 mutants (G93A, G85R, and A4V) were spotted onto a nitrocellulose membrane and blotted with misfolded SOD1 antibodies B8H10 and AMF7-63 under native conditions. SOD1 proteins that are properly folded in a native structure have both copper and zinc bound as well as an intact (oxidized) disulfide bond between Cys57 and Cys146, and are referred to as holo SOD1. Extended incubation of this protein in ambient conditions can generate misfolded or low levels of aggregated holo protein [37]. Note, as recombinant holo SOD1^WT^ aggregates poorly, buffer was applied to the membrane at that position (dashed box). Protein lacking both metal cofactors is referred to as apo SOD1. A reduced apo form of the protein also lacks the crucial Cys57-Cys146 disulfide bond. For all mutations, both AMF7-63 and B8H10 had an increased preference for apo and apo reduced proteins (Fig. 6a). Fully denatured protein served as a positive control.

To determine if apo SOD1 mutants had a preferential association with isolated mitochondria, we performed an in vitro mitochondrial binding assay. Briefly, using siliconized tubes, recombinant human SOD1 proteins were incubated with non-transgenic spinal cord mitochondria, and after washing away unbound protein, mitochondria were recovered and analyzed by western blot for the presence of human SOD1. Recombinant SOD1^G93A^ protein showed an increased binding to mitochondria compared to SOD1^WT^ protein. Treatment with ethylenediamine tetraacetic acid (EDTA), to chelate the metal cofactors of SOD1, resulted in significantly increased binding of SOD1^G93A^ (Fig. 6b, c). SOD1^WT^ displayed a trend toward increased binding to mitochondria following treatment with EDTA (Fig. 6b, c). Treatment with hydrogen peroxide, previously published to oxidize SOD1 [7], did not significantly affect the ability of either recombinant wild-type or mutant SOD1 to associate with mitochondria (Fig. 6b, c). Taken together, misfolded SOD1 antibodies B8H10 and AMF7-63 preferentially detect apo and apo/reduced misfolded SOD1, and this form of mutant SOD1 has an increased association with mitochondria in vitro.

## Discussion

### Misfolded SOD1 specific antibodies recognize distinct non-native SOD1 conformers

In the literature, there are numerous reports of conformational antibodies detecting misfolded SOD1 in various models, tissues and via different methodologies yielding sometimes contradictory conclusions and/or generalizations. We hypothesized that these disparate results could be attributed to differences in the selectivity of these reagents for misfolded SOD1, especially if one considers that “misfolded SOD1” is comprised of more than one species. Thus, we performed a comprehensive comparison of six different antibodies in a single genetic rodent model of ALS using multiple approaches. We find that misfolded SOD1-specific antibodies partition into distinct patterns with A5C3, B8H10, C4F6 and D3H5 antibodies predominantly labeling misfolded SOD1 in motor neurons and numerous puncta within the neuropil. In contrast, the DSE2-3H1 and AMF7-63 antibodies labeled an extensive fibrillar network localized to motor neuron cell bodies, axons, and dendrites. Fibrils are a subset of aggregates composed of β-sheets observed in many neurodegenerative diseases [47, 48]. Whether SOD1 forms fibrils in SOD1-mediated FALS cases, remains controversial [16, 49]. However, inclusions found in the spinal cords of mutant SOD1 animal models contain fibrils that stain positive for Thioflavin T, a molecule that fluoresces upon binding to β-sheets [50, 51]. Interestingly, fibrils have the propensity to seed aggregation in vitro [47], and apo reduced wild-type and mutant SOD1 readily form fibrils in vitro [51]. Moreover, injection of spinal cord homogenates from mice overexpressing wild-type or mutant SOD1 into naïve animal heterozygous YFP-SOD1^G85R^ led to transmission of motor neuron disease and interestingly, different abundances and localizations of SOD1 inclusions and fibrils. These findings suggest that different SOD1 mutants or non-native species may differ both in their ability to “seed” further SOD1 aggregates and the properties of such aggregates [29].

Misfolded SOD1 conformation-specific antibodies may be especially useful at detecting distinct non-native forms of SOD1 and aid in dissecting which species contribute to pathology and potentially help to define the mechanisms implicated. Our work finds that A5C3, AMF7-63 and B8H10-misfolded SOD1 localize to mitochondria whereas as C4F6 does not. Interestingly, although C4F6 and B8H10 were raised against the same immunogen (full length apo SOD1^G93A^ protein), the locations of the epitopes are distinct. The C4F6 epitope is centralized around the G93A mutation (encoded in exon 4) [52], while the B8H10 epitope has been grossly mapped to the loop region encoded by exon 3 [5]. It is noteworthy that these two epitopes are located on opposite sides (~180°) of the three-dimensional structure of the SOD1 protein [5]. That only a subset of neurons carried both epitopes recognized by B8H10 and AMF7-63 whereas other neurons were labeled with only one of these antibodies within the same animals strongly supports that there are indeed multiple non-native misfolded SOD1 conformers in vivo. Moreover, we clearly demonstrate that currently available antibodies represent powerful tools differentiating these conformers that could be used to address the impact of distinct misfolded SOD1 conformers on neuronal properties.

### AMF7-63-reactive misfolded SOD1 correlates with mitochondrial dysfunction

That several misfolded SOD1-reactive conformers converge at the mitochondria highlights mitochondrial dysfunction as an important disease mechanism in ALS. To date, misfolded SOD1 antibodies SEDI [10], DSE2-3H1 [11, 20], A5C3 [11] B8H10 [13] and AMF7-63 (this report) detect misfolded SOD1 at the surface of spinal cord mitochondria. Importantly, in the same spinal cord, AMF7-63- and B8H10-reactive misfolded SOD1 conformers were detected both separately and together on distinct mitochondrial subpopulations again supporting potentially distinct impacts of different SOD1 conformers on mitochondria.

AMF7-63^+^ mitochondria have increased size/volume compared to B8H10^+^ mitochondria, and exhibit a trend toward elevated superoxide production. However, separation into discrete subpopulations, AMF7-63^+^B8H10^−^, AMF7-63^+^B8H10^+^, or B8H10^+^AMF7-63^−^ mitochondria yielded no significant differences between the groups in terms of mitochondrial size/volume, although AMF7-63^+^ and AMF7-63^+^B8H10^+^ showed a trend toward increased volume (data not shown). That the AMF7-63^+^ and B8H10^+^ mitochondrial subpopulations demonstrate differences in mitochondrial size/volume suggest that these antibodies recognize distinct misfolded species that potentially elicit disparate degrees of damage, with AMF7-63 reactive misfolded SOD1 having increased potency. The misfolded SOD1 antibody DSE2-3H1 detects misfolded SOD1 interacting with Voltage-dependent anion channel 1 (VDAC1) [20], a mitochondrial outer membrane protein important for ion homeostasis [53]. It is reported that recombinant mutant SOD1 inhibits VDAC1 conductance in a reconstituted lipid bilayer [20]. Another group, focused on mutant but not misfolded SOD1, reports that the interaction of mutant SOD1 with B-cell lymphoma 2 (Bcl-2) and corresponding exposure of the pro-apoptotic BH3 domain is necessary for Bcl-2 to alter VDAC1 permeability [54]. Our data does not address whether misfolded SOD1 (DSE2-3H1 or B8H10-reactive) interacts with Bcl-2. However, B8H10-reactive misfolded SOD1 and the pro-apoptotic form of Bcl-2 preferentially accumulate on the same mitochondria [13], but this is not indicative of a direct interaction. Furthermore, a portion of B8H10^+^ mitochondria also contain AMF7-63-reactive SOD1 on their surface. Therefore, DSE2-3H1-reactive SOD1 could have an increased association with the pro-apoptotic Bcl-2/VDAC1 complex, resulting in altered mitochondrial ion homeostasis. Future knowledge of the interactome of each misfolded SOD1 conformer may provide insight into the possible differences in toxicity elicited by AMF7-63 and B8H10-reactive misfolded SOD1.

We speculated that DSE2-3H1/AMF7-63-reactive misfolded SOD1 may be prone to aggregation, as fibrils are composed of insoluble, ordered oligomeric chains [55]. However, both B8H10 and AMF7-63 (and DSE2-3H1) labeled aggregates in spinal cord homogenates and isolated mitochondria. Therefore, the increases in mitochondrial size/volume elicited by AMF7-63-reactive misfolded SOD1 cannot be due solely to its participation in aggregate formation at the mitochondrial surface. We cannot exclude the possibility that AMF7-63-reactive misfolded SOD1 is included in aggregates of differing size/properties compared to the B8H10-reactive conformer or that the solubility of these two forms of misfolded SOD1 may differ so as to account for the increased toxicity. C4F6-reactive misfolded SOD1 is not detected in aggregates by this assay, consistent with reports that this antibody recognizes a soluble form of misfolded SOD1 [31, 56].

There is considerable debate over whether SOD1 monomers [17], oligomers [57] or large aggregates [58] mediate toxicity. A caveat to these studies is they have focused on cytosolic SOD1. Mitochondria are vulnerable to proteotoxic stress [59], particularly aggregated proteins [60] and thus, have developed multiple layers of quality control mechanisms to combat this form of stress [61]. Mutant SOD1 has been reported to form aggregates in the matrix of brain mitochondria from ALS animal models [62] and at the surface of mitochondria of cells over-expressing mutant SOD1 [63]. Whether these internal- or surface-localized aggregates contain misfolded SOD1 or cause mitochondrial dysfunction was not determined. However, several recent studies suggest that aggregated SOD1 can perturb mitochondrial membrane integrity in vitro [64, 65]. Our results highlight that multiple misfolded SOD1 conformational antibodies detect misfolded protein, some of which is found in an aggregated form, at the surface of mitochondria. Furthermore, the presence of misfolded SOD1 coincides with disruptions in mitochondrial volume and superoxide production, reinforcing that mitochondria are a *bona fide* target of SOD1 toxicity.

### Demetallated SOD1 is preferentially detected by misfolded SOD1-specific antibodies AMF7-63 and B8H10

Although broadly considered as a cytosolic protein, a small portion of SOD1 is localized to the mitochondrial intermembrane space (IMS) in normal physiological conditions [66]. In order for SOD1 to be imported into mitochondria, it must be in its apo reduced form [67]. Given this, a pool of apo SOD1 at the mitochondrial surface is expected. Interestingly, in our in vitro mitochondrial binding assay, apo SOD1 readily associated with the outer mitochondrial membrane. Import of mitochondrial substrates is slowed in spinal cord mitochondria from SOD1^G93A^ [27], and the regulation of mutant SOD1s import into mitochondria is altered [68], therefore apo mutant SOD1 en route to the IMS may be accumulating at the outer mitochondrial membrane and disturbing normal mitochondrial physiology. Both AMF7-63 and B8H10 detected recombinant apo and apo reduced SOD1 more readily than recombinant holo SOD1.

## Conclusions

**Fig. 7:**
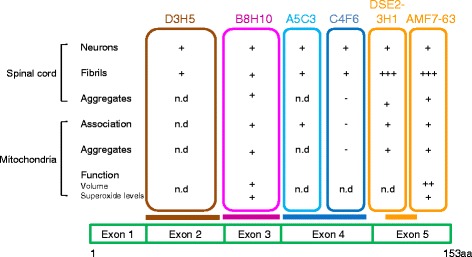
Summary of misfolded SOD1 antibody characteristics. Attributes of various misfolded SOD1 antibodies in spinal cords (presence in neurons, fibrils and aggregates) and isolated spinal cord mitochondria (outer mitochondrial membrane association, presence in aggregates, correlation with damage). Epitopes to misfolded SOD1 antibodies used in this study are grossly mapped to the encoding regions. +, positive finding; −, negative finding; n.d, not determined

Conformational antibodies targeted to misfolded SOD1 show promise not only as therapeutics for ALS, but also as valuable tools with which to probe the mechanisms of misfolded SOD1 toxicity. These antibodies have revealed that multiple non-native species of misfolded SOD1 exist to contribute to motor neuron degeneration, possibly via distinct mechanisms [31, 69]. Our study further supports this premise and highlights that variable potency/toxicity of different SOD1 species is possible even when only one SOD1 mutation is present (Fig. 7). Furthermore, we identify the mitochondria as a target of several of these misfolded SOD1 conformers. This finding may have profound implications for therapeutics aimed at neutralizing misfolded SOD1.

## Additional files

Additional file 1: Figure S1. Misfolded SOD1 specific antibodies do not label SOD1^WT^. A) Lumbar spinal cord sections of a symptomatic SOD1^G93A^ rat and age-matched SOD1^WT^ were labeled with misfolded SOD1 specific antibodies A5C3, B8H10, C4F6, D3H5, DSE2-3H1, AMF7-63 and SEDI (green). B) No non-specific labeling as determined by IgG controls (mouse and rabbit), or incubation with secondary antibody alone, was detected. (PPTX 2948 kb) Additional file 2: Figure S2. Misfolded SOD1 antibody AMF7-63 specifically identifies mutant SOD1 in spinal cord but not liver from SOD1^G93A^ rats. The capacity for AMF7-63 to detect misfolded SOD1 in homogenates or isolated mitochondria from spinal cords and livers was assayed by immunoprecipitation. Rabbit IgG (IgG) serves as control. Input is 10 μg of homogenate or isolated mitochondria. From top to bottom bands correspond to non-specific (ns), human (hSOD1) and rat (rSOD1) SOD1. (PPTX 562 kb) Additional file 3: Figure S3. Misfolded SOD1 antibody B8H10 specifically identifies misfolded SOD1 on the surface of isolated mitochondria from spinal cord but not liver of SOD1^G93A^ rats. A) Immunolabeling of isolated spinal cord and liver mitochondria with misfolded SOD1 antibody B8H10 from symptomatic SOD1^G93A^ rats and controls (age-matched SOD1^WT^ and non-transgenic rats) by flow cytometry. Misfolded SOD1 positive labeling is determined by comparing to isotype control (mouse IgG1) of SOD1^G93A^ sample. Percentage of misfolded SOD1^+^ events is shown for each tissue and genotype in a representative sample. C) Quantification of B8H10^+^ events in spinal cord (blue circles) or liver (black squares) of symptomatic SOD1^G93A^ rats, and age- matched SOD1^WT^ and non-transgenic rats. Data is represented as percent of misfolded B8H10^+^ mitochondria (mean ± SEM). *** *P* < 0.001, *n* = 3 animals per genotype per tissue. (PPTX 253 kb)

## Abbreviations

ALS: Amyotrophic Lateral Sclerosis
Bcl-2: B-cell lymphoma 2
ChAT: choline acetyltransferase
EDTA: ethylenediamine tetraacetic acid
FALS: Familial Amyotrophic Lateral Sclerosis
FSC: forward side scatter
IMS: intermembrane space
MAP2: microtubule associated protein 2
MFI: mean fluorescence intensity
MIF: macrophage inhibitory factor
MTG: Mitotracker Green
SALS: Sporadic Amyotrophic Lateral Sclerosis
SOD1: superoxide dismutase 1
VDAC1: voltage-dependent anion channel
