# Supplementary material for: ALS-linked misfolded SOD1 species have divergent impacts on mitochondria
Source: Acta Neuropathol Commun. 2016 Apr 27;4:43. doi: 10.1186/s40478-016-0313-8 (PMC4847257; doi:10.1186/s40478-016-0313-8)
Supplement: Additional file 1: Figure S1. — Misfolded SOD1 specific antibodies do not label SOD1WT. A) Lumbar spinal cord sections of a symptomatic SOD1G93A rat and age-matched SOD1WT were labeled with misfolded SOD1 specific antibodies A5C3, B8H10, C4F6, D3H5, DSE2-3H1, AMF7-63 and SEDI (green). B) No non-specific labeling as determined by IgG controls (mouse and rabbit), or incubation with secondary antibody alone, was detected. (PPTX 2948 kb) [file 40478_2016_313_MOESM1_ESM.pptx]

## Slide 1
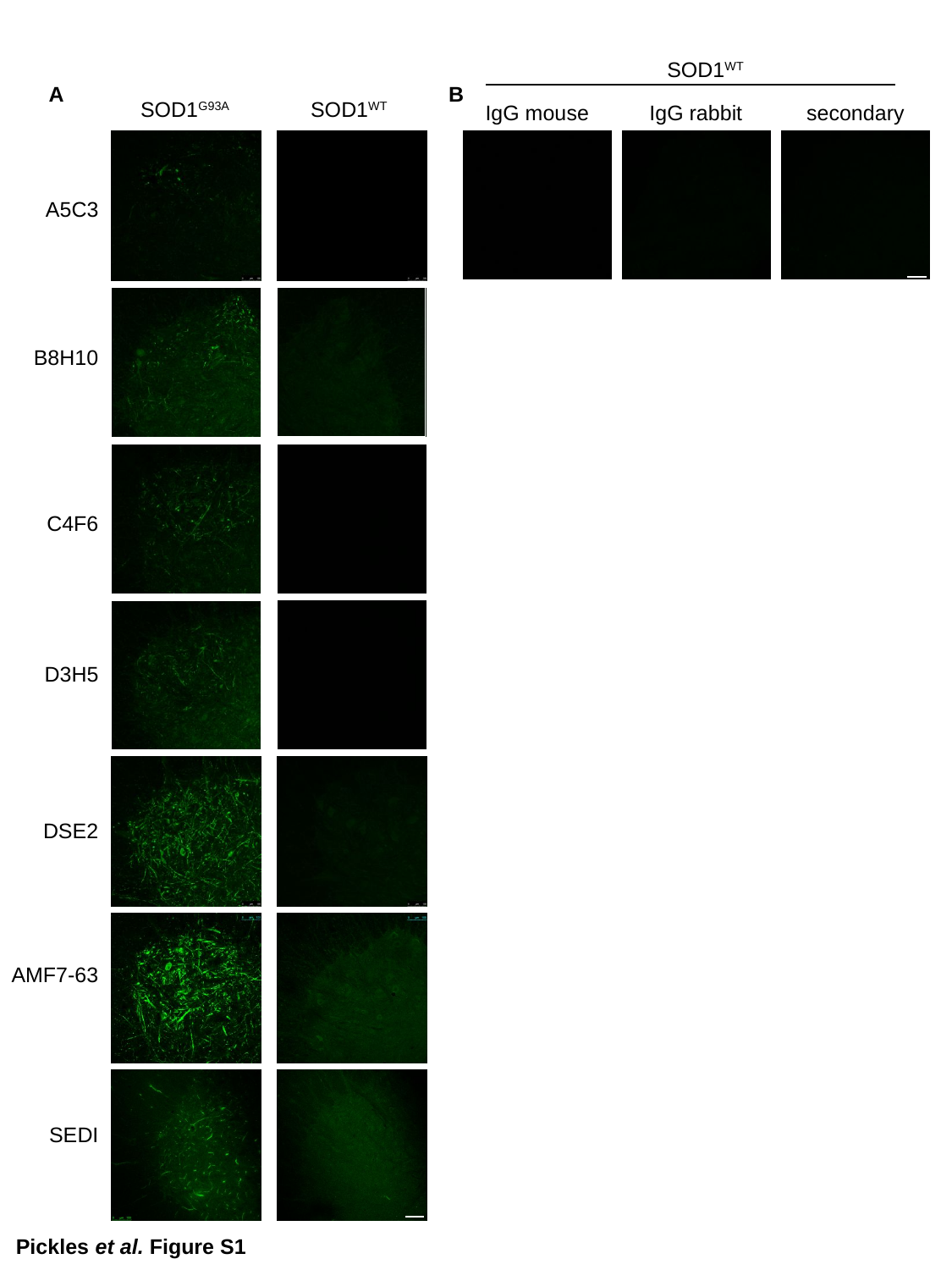

SOD1WT
IgG mouse
IgG rabbit
secondary
A
B
SOD1G93A
SOD1WT
A5C3
B8H10
C4F6
D3H5
DSE2
AMF7-63
SEDI
Pickles et al. Figure S1
