# Supplementary material for: ALS-linked misfolded SOD1 species have divergent impacts on mitochondria
Source: Acta Neuropathol Commun. 2016 Apr 27;4:43. doi: 10.1186/s40478-016-0313-8 (PMC4847257; doi:10.1186/s40478-016-0313-8)
Supplement: Additional file 2: Figure S2. — Misfolded SOD1 antibody AMF7-63 specifically identifies mutant SOD1 in spinal cord but not liver from SOD1G93A rats. The capacity for AMF7-63 to detect misfolded SOD1 in homogenates or isolated mitochondria from spinal cords and livers was assayed by immunoprecipitation. Rabbit IgG (IgG) serves as control. Input is 10 μg of homogenate or isolated mitochondria. From top to bottom bands correspond to non-specific (ns), human (hSOD1) and rat (rSOD1) SOD1. (PPTX 562 kb) [file 40478_2016_313_MOESM2_ESM.pptx]

## Slide 1
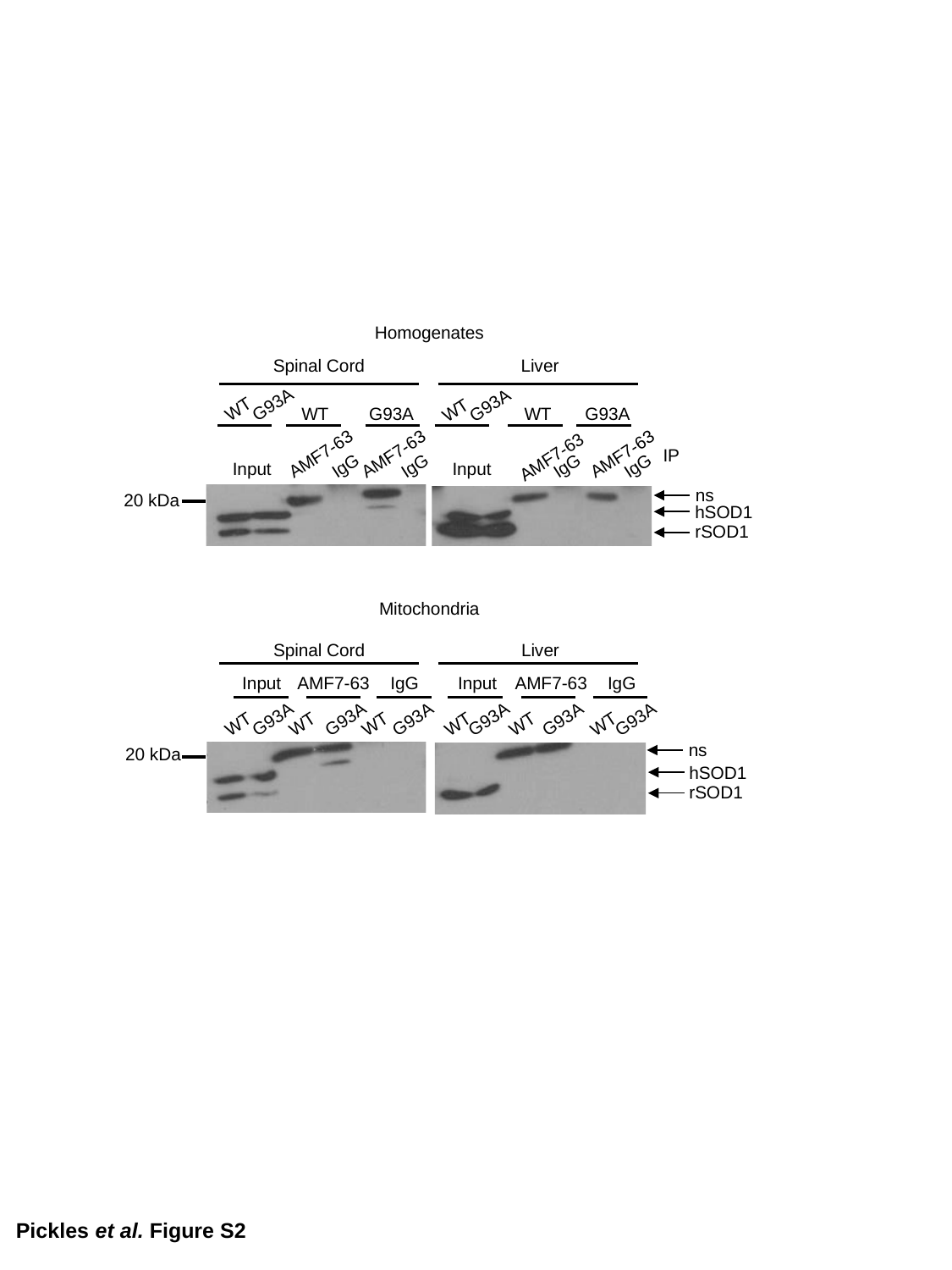

Homogenates
Spinal Cord
Liver
WT
G93A
AMF7-63
IgG
AMF7-63
IgG
IgG
AMF7-63
IgG
AMF7-63
WT
G93A
WT
G93A
Input
Input
hSOD1
rSOD1
20 kDa
WT
G93A
IP
 ns
Mitochondria
Spinal Cord
Liver
Input
AMF7-63
IgG
Input
AMF7-63
IgG
WT
G93A
WT
G93A
WT
G93A
WT
G93A
WT
G93A
WT
G93A
hSOD1
rSOD1
20 kDa
 ns
Pickles et al. Figure S2
