# Supplementary figures and images for: ALS-linked misfolded SOD1 species have divergent impacts on mitochondria
Source: Acta Neuropathol Commun. 2016 Apr 27;4:43. doi: 10.1186/s40478-016-0313-8 (PMC4847257; doi:10.1186/s40478-016-0313-8)

## Slide 1
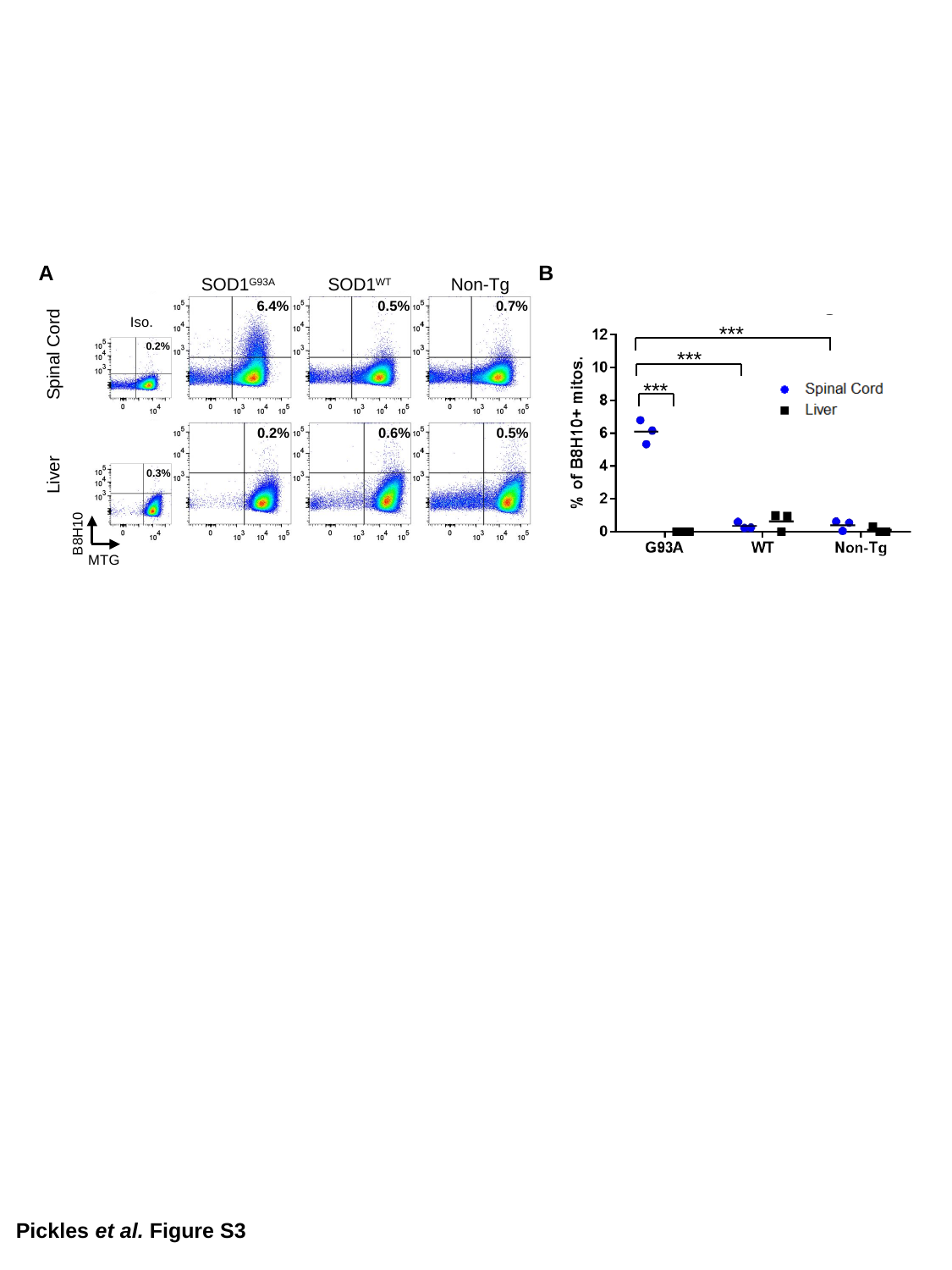

A
SOD1G93A
SOD1WT
Non-Tg
Iso.
Spinal Cord
Liver
B8H10
MTG
6.4%
0.5%
0.7%
0.2%
0.2%
0.6%
0.5%
0.3%
B
***
***
***
Pickles et al. Figure S3

Supplement: Additional file 3: Figure S3. — Misfolded SOD1 antibody B8H10 specifically identifies misfolded SOD1 on the surface of isolated mitochondria from spinal cord but not liver of SOD1G93A rats. A) Immunolabeling of isolated spinal cord and liver mitochondria with misfolded SOD1 antibody B8H10 from symptomatic SOD1G93A rats and controls (age-matched SOD1WT and non-transgenic rats) by flow cytometry. Misfolded SOD1 positive labeling is determined by comparing to isotype control (mouse IgG1) of SOD1G93A sample. Percentage of misfolded SOD1+ events is shown for each tissue and genotype in a representative sample. C) Quantification of B8H10+ events in spinal cord (blue circles) or liver (black squares) of symptomatic SOD1G93A rats, and age- matched SOD1WT and non-transgenic rats. Data is represented as percent of misfolded B8H10+ mitochondria (mean ± SEM). *** P < 0.001, n = 3 animals per genotype per tissue. (PPTX 253 kb) [file 40478_2016_313_MOESM3_ESM.pptx]
